# Natural Products as a Source for New Leads in Depression Treatment

**DOI:** 10.1155/2022/9791434

**Published:** 2022-01-13

**Authors:** Ke Ma, Zulqarnain Baloch, Fengbiao Mao

**Affiliations:** ^1^College of Traditional Chinese Medicine, Shandong University of Traditional Chinese Medicine, Jinan 250355, China; ^2^Faculty of Life Science and Technology, Kunming University of Science and Technology, Kunming, Yunnan 650500, China; ^3^Department of Pathology, University of Michigan, Ann Arbor, MI 48109, USA

Depression is a mental health disorder, which varies from mild to severe changes in mood and affects physical, mental, and behavioral health. Recent evidence indicates that depressive disorders may represent an interactive matrix of reciprocally interactive pathophysiology influenced by various factors like gene or environment. The sustained stress to the individuals with genetic susceptibility leads to the deficits of HPA axis, imbalanced monoamine/cytokine, decreased neurogenesis, and the altered dynamics of connectivity in the reward and motivational circuitry. These mechanisms reduce neuroplasticity and impair the functional integrity of regulation neurocircuitry. Therefore, preventive and therapeutic strategies have been considered to avoid and treat this disease. In this context, the drugs regulating neurotransmitters, including selective serotonin reuptake inhibitors, serotonin and norepinephrine reuptake inhibitors, and tricyclic and monoamine oxidase inhibitors, are the most commonly prescribed antidepressants. However, these antidepressants only have relative success and can originate several side effects, particularly in chronic use. For these reasons, over the years, researchers have been searching for alternative therapeutic strategies, particularly involving the use of natural products.

In this Special Issue, we invited investigators to contribute the latest original research and review articles investigating in vitro, in vivo, nonclinical, and clinical/translational studies addressing the use of traditional Chinese medicine formula, Chinese herbs, and natural products, as either extracts or isolated compounds, in depression treatment. In this Special Issue, six original research articles and two review articles were published regarding the natural products as a source for new leads in depression treatment.

The research article by Y. Choi et al. demonstrated the potential of *Tetragonia tetragonioides* (TTK) in treating depressive symptoms and the alterations in the brain of the animal model of depression. TTK protected the mouse brain glial loss in the prefrontal cortex induced by a gliotoxin, L-AAA. It is suggested that TTK may be one of the potential candidates for treating depression. Further research studies are required to understand the effect of TTK on depressive disorders.

The research article by H. Zhu et al. reported Xiaoyaosan can improve depressive-like behavior in rats by suppressing the activation of the TLR4/NLRP3 inflammasome signaling pathway, thereby inhibiting immunoinflammatory activation and reducing the levels of inflammatory cytokines in the colon. The observed improvement in depressive symptoms may have resulted from the downregulation of the levels of TLR4, MyD88, NF-*κ*B-p65, TAK1, IRAK1, TRAF6, and NLRP3 inflammasome-related NLRP3, ASC, and caspase-1 proteins, leading to the subsequent downregulation of the levels of the inflammatory cytokines IL-6, IL-1*β*, and TNF-*α*.

The research results from M. Qian et al. showed Chaihu Shugan San was superior to fluoxetine in regulating the appearance and HPA axis function of model rats. In addition, Chaihu Shugan San and fluoxetine have similar effects in improving blood theology, and both can alleviate the hypercoagulable state of blood via the platelet 5-hydroxytryptamine receptor 2A (5-HT2A) pathway in rats of depression. It was also observed that Chaihu Shugan San can improve the blood state of depressed rats by restoring liver coagulation-anticoagulation balance and endothelium-related functions.

The research article by M. Jin et al. *So-Ochim-Tang-Gamibang* (SOCG) controlled the levels of HPA axis hormones in a rat model of chronic stress-induced depression. SOCG suppressed the stress-induced increase of the circulating plasma levels of the HPA axis hormones, decreased the ACTH and CRH levels in the pituitary and hypothalamus, respectively, and increased the hippocampal expression of GR, which is an important receptor for the GC hormone. In SOCG-treated depressed rats, an upregulation of the hippocampal expression of the HPA axis-related signaling molecules CREB and ERK was observed; this may lead to the increase of BDNF expression.

The research article by J. Li et al. showed that traditional Chinese medicine (TCM) as a complementary therapy combined with WM was accepted by more than half of the patients with postpartum depression (PPD) and it potentially could lead to a reduced medical expenditure in treating the disease. The study also discussed the associated sociodemographic and medical factors regarding TCM use of PPD patients. The results of our study may be helpful to clinical practitioners as well as health-policy decision-makers while considering the integration of TCM with western medicine in patients with PPD.

The research article by B. Yan et al. suggested acupuncture has a promising application prospect due to its unique advantages for the treatment of chronic pain with depression comorbidity, which can be used in patients suffering from some certain chronic pain with depression comorbidity with poorer response to the conventional medication or suffering from serious side effects. High-quality RCTs are needed to support the current clinical application of acupuncture for the treatment of chronic pain with depression comorbidity and to broaden the clinical application.

The review article by B. Shang et al. summarized the clinical symptoms, etiology, pathogenesis, and therapeutic medication of lily disease and modern Yin-deficient internal heat depression and discusses the relationship between them. Furthermore, the relationship between coronavirus disease 2019 (COVID-19) and lily disease was discussed from the etiology, pathogenesis, and treatment. It provides new ideas for the treatment of COVID-19 and the treatment of psychological problems after recovery.

Another review article by S. Munir et al. investigated the bioactive compounds from plants and marine sources that contain the indole moiety, which can serve as potent antidepressants. The prospects of naturally occurring as well as synthetic indole alkaloids for the amelioration of anxiety and depression-related disorders, structure-activity relationship, and their therapeutic prospects have been discussed.

Thus, with contributions from research groups from diverse countries, this Special Issue presented recent experimental findings and reviews on natural and semisynthetic products with relevant potential for the prevention and treatment of depression.

